# Estimates of the Mutation Rate per Year Can Explain Why the Molecular Clock Depends on Generation Time

**DOI:** 10.1093/molbev/msaf069

**Published:** 2025-03-25

**Authors:** Loveday Lewin, Adam Eyre-Walker

**Affiliations:** School of Life Sciences, University of Sussex, Brighton BN1 9QG, UK; School of Life Sciences, University of Sussex, Brighton BN1 9QG, UK

**Keywords:** generation time, mutation rate, evolutionary rate

## Abstract

Rates of molecular evolution are known to vary across species, often deviating from the classical expectation of a strict molecular clock. In many cases, the rate of molecular evolution has been found to correlate to generation time, an effect that could be explained if species with shorter generation times have higher mutation rates per year. We investigate this hypothesis using direct estimates of the mutation rate for 133 eukaryotic species from diverse taxonomic groups. Using a phylogenetic comparative approach, we find a strong negative correlation between mutation rate per year and generation time, consistent across all phylogenetic groups. Our results provide a simple explanation for why generation time plays a pivotal role in driving rates of molecular evolution across eukaryotes.

## Introduction

The concept of a “molecular clock,” whereby DNA sequence evolution proceeds at a constant, clock-like rate, was first introduced in the 1960s (Zuckerkandl and Pauling 1962). The molecular clock was initially heralded as a powerful tool for estimating evolutionary rates and divergence times of species across the tree of life. However, more recent tests of the strict clock hypothesis have revealed that the rate of molecular evolution (measured as the rate at which protein, RNA, or DNA sequences evolve per year) varies widely between species, especially when considering more distantly related lineages (Bousquet et al. 1992; Thomas et al. 2006). This was first discovered by Wu and Li (1985) in a comparison of protein-coding sequences from primates, rodents, and artiodactyls; they found that rodents were about twice as divergent from their common ancestor as primates. Studies have found this variation in rate to be correlated to several aspects of species biology, including body size, longevity, metabolic rate, DNA repair mechanisms, and generation time (Bromham 2002; Nabholz et al. 2008; Smith and Donoghue 2008; Thomas et al. 2010). Among these traits, generation time has emerged as a particularly strong correlate of molecular evolution rates, such that species with short generation times have higher rates of molecular evolution. This effect of generation time on the rate of evolution in both nuclear and mitochondrial DNA has been observed extensively in mammals (Ohta 1993; Bromham et al. 1996; Li et al. 1996; Rowe and Honeycutt 2002; Welch et al. 2008; Sayres et al. 2011), but also in diverse taxa including plants (Smith and Donoghue 2008), invertebrates (Thomas et al. 2010), and bacteria (Weller and Wu 2015).

It is thought that the association between the rate of molecular evolution and generation time is due to a correlation between the mutation rate and generation time. However, until recently, there have been few direct estimates of the mutation rate available to confirm or refute this hypothesis. The advent of next-generation sequencing has enabled direct estimation of mutation rates through parent–offspring sequencing and mutation accumulation experiments, offering an opportunity to test this hypothesis directly. A number of studies comparing the mutation rates of individual mammalian species to humans have observed that species with shorter generation times tend to have higher mutation rates per year (Wu et al. 2020; Bergeron et al. 2021; Wang et al. 2022b). Bergeron et al. (2023) further showed that the relationship between the mutation rate per generation and generation time across species has a positive intercept, implying that a negative correlation between the mutation rate per year and generation time should exist. However, the relationship between the mutation rate per year—the fundamental determinant of rates of molecular evolution—and generation time remains underexplored across a more extensive phylogeny. In this study, we leverage a growing data set of directly estimated mutation rates to investigate whether the mutation rate per year is correlated to generation time across a wide range of species.

## Methods

The germline mutation rate estimates used in this study were primarily compiled from an existing meta-analysis by Wang and Obbard (2023). An additional literature search was performed using Clarivate Web of Science following the search strategy described in Wang and Obbard (2023) for the period from 2022 September 22 to 2024 April 15 to ensure inclusion of more recent data. This yielded an additional 30 estimates and brought the total number of species with genome-wide mutation rate estimates to 156 (supplementary table S1, Supplementary Material online). All estimates were based on sequencing of closely related individuals in pedigrees or mutation accumulation lines.

Generation time estimates for 146 of these species were sourced from the literature (supplementary table S2, Supplementary Material online). In the context of this analysis, the generation time is the average age of parents. Unfortunately, most species lack sufficiently detailed reproductive data to estimate this directly. As a result, most generation time estimates are modeled based on available life history traits such as age at first breeding and reproductive life span (full details of how these estimates were made can be found in supplementary table S2, Supplementary Material online). Although this approach may introduce a level of error into estimates of generation time—particularly for iteroparous species, for which age at first breeding likely underestimates average parental age—indirect generation time estimates should still provide a reasonable approximation for large-scale comparative studies such as this. Prior work has demonstrated that such estimates correlate well with empirical data for species where direct measurements are available (Pacifici et al. 2013), and given the scope and diversity of species included in this study, indirect generation time estimates offer a practical means of addressing gaps in reproductive data while ensuring the feasibility of analyzing broad evolutionary trends.

Mutation rates per year (μ_y_) were subsequently calculated as μ_G_/generation time, where μ_G_ is the mutation rate per site per generation. μ_y_ estimates ranged from 1.51 × 10^−6^ mutations per site per year in *Saccharomyces cerevisiae* down to the lowest estimate of 1.26 × 10^−10^ per year in the snowy owl (supplementary table S2, Supplementary Material online). Since these values vary over orders of magnitude, all analyses following the initial calculation of μ_y_ were performed on log(μ_y_) and log(generation time), though for simplicity we refer to these variables as μ_y_ and generation time, respectively.

Bergeron et al. (2023) note that estimating μ_y_ as μ_G_/generation time may lead to a biased estimate of the mutation rate per year if the average age of parents in the experiment used to determine the mutation rate differs from the generation time in the wild. They propose a method to control for this, but they assume that the number of mutations per site at birth is the same across all species. While this assumption may be reasonable for the vertebrates they have considered, it seems less likely to be the case for our more diverse selection of species. Our estimates are only likely to be substantially biased if the parental age in the experiment differs significantly from the generation time in the wild *and* mutation rates increase substantially with parental age. The available evidence suggests that many mutations occur before birth (Gao et al. 2014; Lindsay et al. 2019), and although there is evidence that the mutation rate increases with parental age in many species (Lindsay et al. 2019; Wang et al. 2022b; Bergeron et al. 2023), in most cases, the slope is shallow relative to the intercept—i.e. in those species that show a parental age effect, the total number of mutations at reproductive age is often dominated by those that occurred before birth, not those that occurred post-puberty.

To test for an association between μ_y_ and generation time, phylogenetic comparative methods were employed to account for the shared evolutionary history between species (Felsenstein 1985). A phylogenetic tree was constructed using https://timetree.org/ (Kumar et al. 2022), and phylogenetic generalized least squares (PGLS) regressions were performed using the R package caper (Orme et al. 2023) to correct for phylogenetic nonindependence when fitting regression lines. Phylogenetic generalized linear mixed models (PGLMM) were performed using the R package phyr (Li et al. 2020). Thirteen species were lost from our analysis since TimeTree had no data on them or a closely related species; this meant that the following analyses were ultimately performed on a final set of 133 species.

To investigate whether the relationship between μ_y_ and generation time might be due to estimation error in the generation time, we performed a simulation in which we assumed that the mutation rate per year was independent of the generation time. We sampled log(μ_y__true) and log(GT_true) from normal distributions, along with a normally distributed error term. We calculated log(μ_G__true) = log(μ_y__true) + log(GT_true) and log(GT_obs) = log(GT_true) + *e*, where *e* is the error in the generation time. The estimated mutation rate per year is then log(μ_y__obs) = log(μ_G__true) − log(GT_obs). We considered the regression between log(μ_y__obs) and log(GT_obs) for data sets of 133 species. We repeated the regression 100 times for each set of parameters varying the error variance across simulations. log(GT_obs) was constrained to equal the variance observed in the data we have analyzed.

## Results and Discussion

**Fig. 1.**
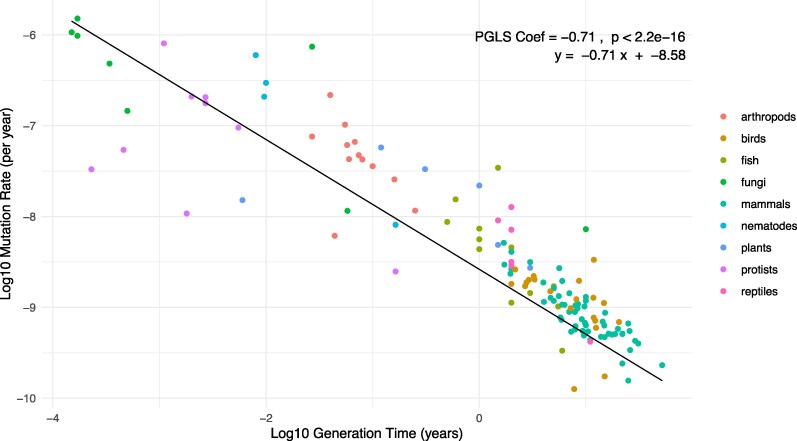
PGLS regression of mutation rate per year (μ_y_) on generation time (in years) on a log10 scale. After controlling for phylogenetic inertia, mutation rate per year remains significantly negatively correlated to generation time. Adjusted *R*^2^ = 0.50. Standard error associated with the slope = 0.062.

Using our data set of 133 species, we find that the mutation rate per year (μ_y_) is significantly negatively correlated to generation time (PGLS slope = −0.71, *P* < 0.01; Fig. 1; supplementary fig. S2, Supplementary Material online), such that species with shorter generation times are estimated to have higher mutation rates per year. The slope of the regression implies that doubling the generation time will reduce the mutation rate per year by approximately 39%.

This relationship appears to be consistent across diverse phylogenetic groups. Classifying species into nine arbitrary taxonomic groups, we find that the relationship between μ_y_ and generation time is negative in all cases, with many groups showing statistically significant correlations (Table 1). To investigate whether the nature of the relationship differed among groups, we used a PGLMM of μ_y_ on generation time, incorporating phylogenetic group as a fixed effect. This model allowed us to test whether the intercept and slope of the relationship between μ_y_ and generation time vary across groups, while accounting for the nonindependence of species due to their phylogenetic relationships by specifying a phylogenetic tree as a covariance structure for the species as a random effect term. In our analysis, we found no evidence that adding phylogenetic group, or an interaction between the slope and phylogenetic group, improved the fit of the model. While some groups contained relatively few species, potentially limiting the power of our analyses, we detected no significant difference in the intercept of the relationship when comparing the two largest groups, mammals and birds, using a two-sample *t*-test (*P* = 0.33); likewise, we found no significant difference in the slopes (*P* = 0.66).

**Table 1:** Summary of PGLS regressions of mutation rate per year (μ_y_) on generation time (in years) within individual phylogenetic groups

Phylogenetic group	Number of species	PGLS slope	*P*-value	Adjusted *R*^2^
Arthropods	12	−0.84	0.0980	0.17
Birds	20	−0.63	0.0242^a^	0.21
Fish	12	−1.20	0.0027^b^	0.57
Fungi	8	−0.57	0.0057^b^	0.70
Mammals	56	−0.80	<2.2e-16^b^	0.76
Nematodes	4	−1.31	0.0071^b^	0.98
Plants	6	−0.26	0.3160	0.06
Protists	9	−0.47	0.1703	0.14
Reptiles	6	−1.67	0.0261^a^	0.69

^a^ *P* < 0.05. ^b^*P* < 0.01.

The negative relationship identified between μ_y_ and generation time could potentially be a consequence of error in the generation time estimates, which are only approximations for species with complex life histories or incomplete ecological data. The fact that μ_y_ is estimated as the mutation rate per generation divided by generation time means that sampling error in the generation time estimates could generate an artifactual negative correlation between the two. To investigate this, we ran a simulation in which we assumed that μ_y_ was independent of generation time, with both generation time values and the error in these values being sampled from independent normal distributions. We find that the error variance in generation time would need to be approximately 2.2 times greater than the systematic variance in generation time to produce the observed slope (supplementary fig. S1, Supplementary Material online). A similar result can also be derived analytically (see supplementary text S1, Supplementary Material online). Given that it is unrealistic for the error in our generation time estimates to substantially exceed the systematic variation in generation time, we conclude that there is a genuine negative relationship between μ_y_ and generation time.

We have shown that μ_y_ is negatively correlated to generation time. This provides an intuitive explanation as to why the rate of molecular evolution is correlated to generation time, as we expect this rate to depend on the mutation rate under almost every model of molecular evolution. However, *why* μ_y_ is negatively correlated to generation time is less clear. Mutations can occur due to errors in DNA replication or from DNA damage that is either unrepaired or incorrectly repaired prior to the next round of replication. Although the relative contributions of these processes to the overall level of mutation remain unclear and actively debated (Gao et al. 2016; Wang et al. 2020; Wu et al. 2020; Hahn et al. 2023), there are various models incorporating different mutational processes that could account for the negative relationship between μ_y_ and generation time.

One simple model is that the mutation rate during early gametogenesis is higher than in later stages. If early rounds of cell division are significantly more mutagenic, regardless of whether those mutations are replicative or nonreplicative in origin, then μ_y_ will decrease with increasing generation time. First, it has been shown in both *Drosophila* (Gao et al. 2011, 2014) and mice (Lindsay et al. 2019) that the mutation rate is elevated during early germ cell development, with 24% of all germline mutations in mice found to occur during the first three cell divisions. Second, as several authors have noted, the intercept of a regression of the number of mutations against parental age typically has a positive intercept, implying that a significant number of mutations have accumulated before birth (Lindsay et al. 2019; Wang et al. 2022b; Bergeron et al. 2023). If this pattern holds across species, then organisms with shorter generation times will have higher μ_y_ due to a higher frequency of mutagenic developmental cycles per unit time.

An alternative hypothesis that is commonly invoked to explain the generation time effect is that organisms with shorter generation times have a faster rate of cell division and therefore accumulate more mutations due to replication errors (Li et al. 1996). However, evidence in mammals increasingly supports a decoupling of the mutational burden from cell division. Firstly, a modest but significant maternal age effect has been observed in humans, with older mothers found to transmit more germline mutations despite the absence of ongoing cell division in the female germline (Goldmann et al. 2016; Wong et al. 2016; Jónsson et al. 2017). Secondly, the paternal mutation bias in mammals—whereby most germline mutations are transmitted from the father—has been found to be present by the age of puberty despite similar numbers of cell divisions occurring in the male and female germlines up until this point (Drost and Lee 1995; Forster et al. 2015; Gao et al. 2019). Finally, the degree of male mutation bias appears to be relatively consistent across species with very different generation times (Lindsay et al. 2019; Wu et al. 2020; Wang et al. 2022a), casting further doubt on the assumption that germline mutations track cell divisions. In the absence of a clear link between cell division rates and mutation rates, replication-driven mutations cannot fully account for the negative correlation between μ_y_ and generation time.

It could instead be that organisms with shorter generation times experience greater levels of nonreplicative damage, either due to higher rates of lesion formation or more error-prone DNA repair processes. This could explain the observed negative relationship without having to invoke replication-dependent mutations. A similar hypothesis has been proposed to account for the male mutation bias in mammals, with the suggestion that males have less robust DNA repair and/or protection mechanisms than females (de Manuel et al. 2022; Hahn et al. 2023). An analogous model whereby species with shorter generation times accumulate more damage-induced mutations due to a reduced capacity to protect their germline could help to explain the observed negative correlation between μ_y_ and generation time. This effect could be complemented by longer generation times inducing stronger selection against “mutator alleles”, such as those reducing the efficiency of DNA repair (Lindsay et al. 2019; Zhu et al. 2024). This heightened selection pressure could help to counteract the weaker purifying selection expected in species with smaller effective population sizes (Lynch 2010), ultimately favoring lower μ_y_ to limit the accumulation of germline mutations within each generation.

As it stands, the timings and absolute contributions of replicative and nonreplicative mutations in the germline remain largely unknown, so we cannot definitively explain the negative correlation between μ_y_ and generation time. However, regardless of the underlying mechanism behind this correlation, it still provides a simple explanation as to why the widely observed relationship between rates of molecular evolution and generation time exists. By identifying systematic correlates of rates of molecular evolution, such as generation time, it becomes possible to increase the flexibility and accuracy of the molecular clock by integrating these variables into relaxed clock models (Lartillot et al. 2016). Overall, these findings highlight the importance of generation time as a predictor of mutation rates, providing valuable insights for refining evolutionary models and improving our understanding of how life history traits shape molecular evolution across the tree of life.

## Supplementary Material

msaf069_Supplementary_Data

## Data Availability

All data and scripts used in this study are available on GitHub at https://github.com/lovedayel/mutation-generation-evolution.

## References

[msaf069-B1] Bergeron LA, Besenbacher S, Bakker J, Zheng J, Li P, Pacheco G, Sinding M-HS, Kamilari M, Gilbert MTP, Schierup MH, et al The germline mutational process in rhesus macaque and its implications for phylogenetic dating. GigaScience. 2021:10(5):giab029. 10.1093/gigascience/giab029. 33954793 PMC8099771

[msaf069-B2] Bergeron LA, Besenbacher S, Zheng J, Li P, Bertelsen MF, Quintard B, Hoffman JI, Li Z, St. Leger J, Shao C, et al Evolution of the germline mutation rate across vertebrates. Nature. 2023:615(7951):285–291. 10.1038/s41586-023-05752-y. 36859541 PMC9995274

[msaf069-B3] Bousquet J, Strauss SH, Doerksen AH, Price RA. Extensive variation in evolutionary rate of rbcL gene sequences among seed plants. Proc Natl Acad Sci U S A. 1992:89(16):7844–7848. 10.1073/pnas.89.16.7844. 1502205 PMC49808

[msaf069-B4] Bromham L . Molecular clocks in reptiles: life history influences rate of molecular evolution. Mol Biol Evol. 2002:19(3):302–309. 10.1093/oxfordjournals.molbev.a004083. 11861889

[msaf069-B5] Bromham L, Rambaut A, Harvey PH. Determinants of rate variation in mammalian DNA sequence evolution. J Mol Evol. 1996:43(6):610–621. 10.1007/BF02202109. 8995058

[msaf069-B6] de Manuel M, Wu FL, Przeworski M. A paternal bias in germline mutation is widespread in amniotes and can arise independently of cell division numbers. eLife. 2022:11:e80008. 10.7554/eLife.80008. 35916372 PMC9439683

[msaf069-B7] Drost JB, Lee WR. Biological basis of germline mutation: comparisons of spontaneous germline mutation rates among Drosophila, mouse, and human. Environ Mol Mutagen. 1995:25(S2):48–64. 10.1002/em.2850250609. 7789362

[msaf069-B8] Felsenstein J . Phylogenies and the comparative method. Am Nat. 1985:125(1):1–15. 10.1086/284325. 31094602

[msaf069-B9] Forster P, Hohoff C, Dunkelmann B, Schürenkamp M, Pfeiffer H, Neuhuber F, Brinkmann B. Elevated germline mutation rate in teenage fathers. Proc R Soc B Biol Sci. 2015:282(1803):20142898. 10.1098/rspb.2014.2898. PMC4345458 25694621

[msaf069-B10] Gao J-J, Pan X-R, Hu J, Ma L, Wu J-M, Shao Y-L, Ai S-M, Liu S-Q, Barton SA, Woodruff RC, et al Pattern of mutation rates in the germline of *Drosophila melanogaster* males from a large-scale mutation screening experiment. G3 (Bethesda). 2014:4(8):1503–1514. 10.1534/g3.114.011056. 24924332 PMC4132180

[msaf069-B11] Gao J-J, Pan X-R, Hu J, Ma L, Wu J-M, Shao Y-L, Barton SA, Woodruff RC, Zhang Y-P, Fu Y-X. Highly variable recessive lethal or nearly lethal mutation rates during germ-line development of male Drosophila melanogaster. Proc Natl Acad Sci U S A. 2011:108(38):15914–15919. 10.1073/pnas.1100233108. 21890796 PMC3179084

[msaf069-B12] Gao Z, Moorjani P, Sasani TA, Pedersen BS, Quinlan AR, Jorde LB, Amster G, Przeworski M. Overlooked roles of DNA damage and maternal age in generating human germline mutations. Proc Natl Acad Sci U S A. 2019:116(19):9491–9500. 10.1073/pnas.1901259116. 31019089 PMC6511033

[msaf069-B13] Gao Z, Wyman MJ, Sella G, Przeworski M. Interpreting the dependence of mutation rates on age and time. PLOS Biol. 2016:14(1):e1002355. 10.1371/journal.pbio.1002355. 26761240 PMC4711947

[msaf069-B14] Goldmann JM, Wong WSW, Pinelli M, Farrah T, Bodian D, Stittrich AB, Glusman G, Vissers LELM, Hoischen A, Roach JC, et al Parent-of-origin-specific signatures of de novo mutations. Nat Genet. 2016:48(8):935–939. 10.1038/ng.3597. 27322544

[msaf069-B15] Hahn MW, Peña-Garcia Y, Wang RJ. The “faulty male” hypothesis for sex-biased mutation and disease. Curr Biol. 2023:33(22):R1166–R1172. 10.1016/j.cub.2023.09.028. 37989088 PMC11795531

[msaf069-B16] Jónsson H, Sulem P, Kehr B, Kristmundsdottir S, Zink F, Hjartarson E, Hardarson MT, Hjorleifsson KE, Eggertsson HP, Gudjonsson SA, et al Parental influence on human germline de novo mutations in 1,548 trios from Iceland. Nature. 2017:549(7673):519–522. 10.1038/nature24018. 28959963

[msaf069-B17] Kumar S, Suleski M, Craig JM, Kasprowicz AE, Sanderford M, Li M, Stecher G, Hedges SB. TimeTree 5: an expanded resource for species divergence times. Mol Biol Evol. 2022:39(8):msac174. 10.1093/molbev/msac174. 35932227 PMC9400175

[msaf069-B18] Lartillot N, Phillips MJ, Ronquist F. A mixed relaxed clock model. Philos. Trans. R. Soc. B Biol. Sci. 2016:371(1699):20150132. 10.1098/rstb.2015.0132. PMC4920333 27325829

[msaf069-B19] Li D, Dinnage R, Nell LA, Helmus MR, Ives AR. phyr: an r package for phylogenetic species-distribution modelling in ecological communities. Methods Ecol Evol. 2020:11(11):1455–1463. 10.1111/2041-210X.13471.

[msaf069-B20] Li W-H, Ellsworth DL, Krushkal J, Chang BH-J, Hewett-Emmett D. Rates of nucleotide substitution in primates and rodents and the generation–time effect hypothesis. Mol Phylogenet Evol. 1996:5(1):182–187. 10.1006/mpev.1996.0012. 8673286

[msaf069-B21] Lindsay SJ, Rahbari R, Kaplanis J, Keane T, Hurles ME. Similarities and differences in patterns of germline mutation between mice and humans. Nat Commun. 2019:10(1):4053. 10.1038/s41467-019-12023-w. 31492841 PMC6731245

[msaf069-B22] Lynch M . Evolution of the mutation rate. Trends Genet. 2010:26(8):345–352. 10.1016/j.tig.2010.05.003. 20594608 PMC2910838

[msaf069-B23] Nabholz B, Glémin S, Galtier N. Strong variations of mitochondrial mutation rate across mammals—the longevity hypothesis. Mol Biol Evol. 2008:25(1):120–130. 10.1093/molbev/msm248. 17998254

[msaf069-B24] Ohta T . An examination of the generation-time effect on molecular evolution. Proc Natl Acad Sci U S A. 1993:90(22):10676–10680. 10.1073/pnas.90.22.10676. 8248159 PMC47840

[msaf069-B25] Orme D, Freckleton R, Thomas G, Petzoldt T, Fritz S, Isaac N, Pearse W. 2023. caper: comparative analyses of phylogenetics and evolution in R. [accessed 2025 Feb 2]. https://cran.r-project.org/web/packages/caper/index.html

[msaf069-B26] Pacifici M, Santini L, Marco MD, Baisero D, Francucci L, Marasini GG, Visconti P, Rondinini C. Generation length for mammals. Nat. Conserv. 2013:5:89–94. 10.3897/natureconservation.5.5734.

[msaf069-B27] Rowe DL, Honeycutt RL. Phylogenetic relationships, ecological correlates, and molecular evolution within the Cavioidea (Mammalia, Rodentia). Mol Biol Evol. 2002:19(3):263–277. 10.1093/oxfordjournals.molbev.a004080. 11861886

[msaf069-B28] Sayres MAW, Venditti C, Pagel M, Makova KD. Do variations in substitution rates and male mutation bias correlate with life-history traits? A study of 32 mammalian genomes. Evolution. 2011:65(10):2800–2815. 10.1111/j.1558-5646.2011.01337.x. 21967423

[msaf069-B29] Smith SA, Donoghue MJ. Rates of molecular evolution are linked to life history in flowering plants. Science. 2008:322(5898):86–89. 10.1126/science.1163197. 18832643

[msaf069-B30] Thomas JA, Welch JJ, Lanfear R, Bromham L. A generation time effect on the rate of molecular evolution in invertebrates. Mol Biol Evol. 2010:27(5):1173–1180. 10.1093/molbev/msq009. 20083649

[msaf069-B31] Thomas JA, Welch JJ, Woolfit M, Bromham L. There is no universal molecular clock for invertebrates, but rate variation does not scale with body size. Proc Natl Acad Sci U S A. 2006:103(19):7366–7371. 10.1073/pnas.0510251103. 16651532 PMC1464347

[msaf069-B32] Wang RJ, Peña-Garcia Y, Bibby MG, Raveendran M, Harris RA, Jansen HT, Robbins CT, Rogers J, Kelley JL, Hahn MW. Examining the effects of hibernation on germline mutation rates in grizzly bears. Genome Biol Evol. 2022a:14(10):evac148. 10.1093/gbe/evac148. 36173788 PMC9596377

[msaf069-B33] Wang RJ, Raveendran M, Harris RA, Murphy WJ, Lyons LA, Rogers J, Hahn MW. De novo mutations in domestic cat are consistent with an effect of reproductive longevity on both the rate and spectrum of mutations. Mol Biol Evol. 2022b:39(7):msac147. 10.1093/molbev/msac147. 35771663 PMC9290555

[msaf069-B34] Wang RJ, Thomas GWC, Raveendran M, Harris RA, Doddapaneni H, Muzny DM, Capitanio JP, Radivojac P, Rogers J, Hahn MW. Paternal age in rhesus macaques is positively associated with germline mutation accumulation but not with measures of offspring sociability. Genome Res. 2020:30(6):826–834. 10.1101/gr.255174.119. 32461224 PMC7370888

[msaf069-B35] Wang Y, Obbard DJ. Experimental estimates of germline mutation rate in eukaryotes: a phylogenetic meta-analysis. Evol Lett. 2023:7(4):216–226. 10.1093/evlett/qrad027. 37475753 PMC10355183

[msaf069-B36] Welch JJ, Bininda-Emonds OR, Bromham L. Correlates of substitution rate variation in mammalian protein-coding sequences. BMC Evol Biol. 2008:8:53. 10.1186/1471-2148-8-53. 18284663 PMC2289806

[msaf069-B37] Weller C, Wu M. A generation-time effect on the rate of molecular evolution in bacteria. Evolution. 2015:69(3):643–652. 10.1111/evo.12597. 25564727

[msaf069-B38] Wong WSW, Solomon BD, Bodian DL, Kothiyal P, Eley G, Huddleston KC, Baker R, Thach DC, Iyer RK, Vockley JG, et al New observations on maternal age effect on germline de novo mutations. Nat Commun. 2016:7(1):10486. 10.1038/ncomms10486. 26781218 PMC4735694

[msaf069-B39] Wu CI, Li WH. Evidence for higher rates of nucleotide substitution in rodents than in man. Proc Natl Acad Sci U S A. 1985:82(6):1741–1745. 10.1073/pnas.82.6.1741. 3856856 PMC397348

[msaf069-B40] Wu FL, Strand AI, Cox LA, Ober C, Wall JD, Moorjani P, Przeworski M. A comparison of humans and baboons suggests germline mutation rates do not track cell divisions. PLOS Biol. 2020:18(8):e3000838. 10.1371/journal.pbio.3000838. 32804933 PMC7467331

[msaf069-B41] Zhu L, Beichman A, Harris K. Population size interacts with reproductive longevity to shape the germline mutation rate. bioRxiv 570457. 10.1101/2023.12.06.570457, 9 November 2024, preprint: not peer reviewed. PMC12130860 40392851

[msaf069-B42] Zuckerkandl E, Pauling L. Molecular disease, evolution, and genetic heterogeneity. New York: Academic Press; 1962. p. 189–225.

