# Supplementary material for: Estimates of the Mutation Rate per Year Can Explain Why the Molecular Clock Depends on Generation Time
Source: Mol Biol Evol. 2025 Mar 25;42(4):msaf069. doi: 10.1093/molbev/msaf069 (PMC11969216; doi:10.1093/molbev/msaf069)
Supplement: msaf069_Supplementary_Data [file msaf069_supplementary_data.zip › supplementary_materials.pdf]

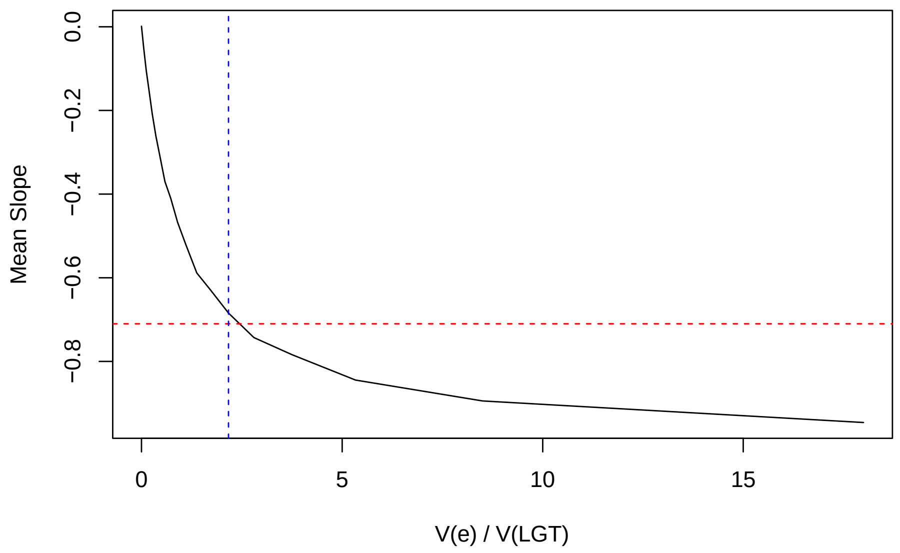

Supplementary figure 1. Simulation results: the ratio of error variance to systematic variance in log(GT) required to produce the observed slope under a model in which the mutation rate per year is uncorrelated to generation time.

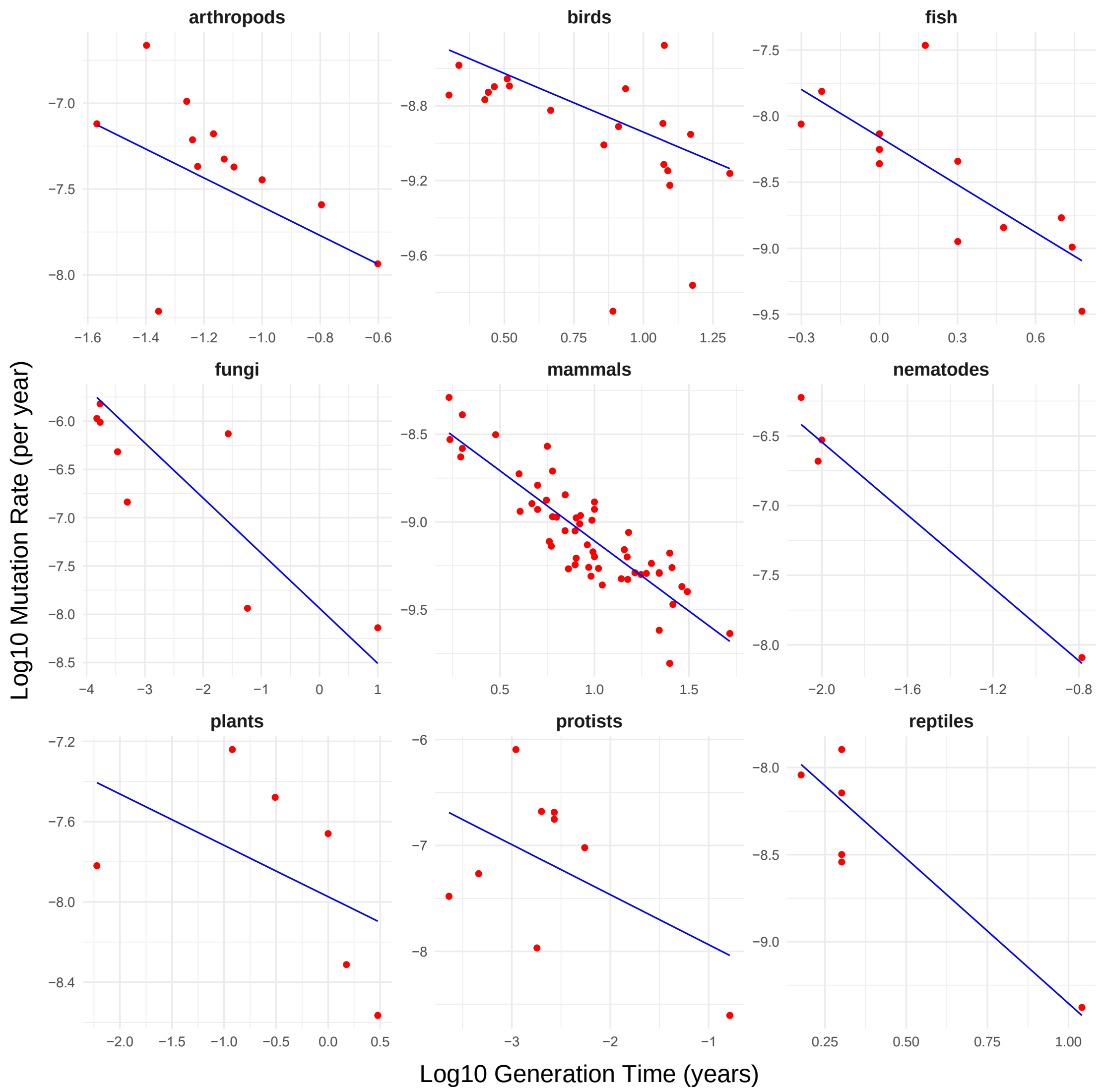

Supplementary figure 2. PGLS regression of mutation rate per year on generation time (in years) on a log10 scale within individual phylogenetic groups.

Supplementary Text

We are interested in the relationship between the mutation rate per year, u_{year}, and generation time, g, and whether the negative relationship between these two is simply due to error in estimating g. Let us rephrase the question in terms of the relationship between the mutation rate per generation, u_{gen}, and g. First, we note that

$$u_{gen} = u_{year} g \quad (1)$$

Hence if we take logs we have

$$\log(u_{gen}) = \log(g) + \log(u_{year}) \quad (2)$$

And hence we expect the relationship between $\log(u_{gen})$ and $\log(g)$ to have a slope of one assuming that u_{year} is uncorrelated to g if g is known without error.

Consider the slope of the relationship between $y = \log(u_{gen})$ and $\mathrm{x} = \log(g)$assuming the generation time is known without error

$$\beta = \frac{Cov(\mathrm{x,y})}{V(x)} = 1 \quad (3)$$

hence $Cov(x, y) = V(x)$. Now consider the slope when we have error in our generation time estimate. The slope can be written as

$$\beta_e = \frac{Cov(x,y)}{V(x)+V(e)} \quad (4)$$

Where *V(e)* is the error variance of $\log(g)$. Noting that $Cov(x, y) = V(x)$ we can write equation 4 as

$$\beta_e = \frac{Cov(x,y)}{V(x)+V(e)} = \frac{V(x)}{V(x)+V(e)} = \frac{1}{1+\alpha} \quad (5)$$

Where $\alpha = V(e)/V(x)$ the ratio of the error variance to the systematic variance in $\log(g)$. We can rearrange equation 5 to get an expression for α in terms of the slope.

$$\alpha = \frac{1-\beta_e}{\beta_e} \quad (6)$$

The observed slope of the relationship between $\log(u_{gen})$ and $\log(g)$ is 0.31 which implies that $\alpha =$ 2.2 in agreement with our simulation. This implies that to explain the negative relationship between $\log(u_{year})$ and $\log(g)$ through sampling error in g, assuming there is no genuine relationship, requires the error variance in $\log(u_{gen})$ to be 2.2x as large as the systematic variance. This seems unrealistic.
